# Levels and Determinants of Inflammatory Biomarkers in a Swiss Population-Based Sample (CoLaus Study)

**DOI:** 10.1371/journal.pone.0021002

**Published:** 2011-06-09

**Authors:** Pedro Marques-Vidal, Murielle Bochud, François Bastardot, Thomas Lüscher, François Ferrero, Jean-Michel Gaspoz, Fred Paccaud, Adrian Urwyler, Roland von Känel, Christoph Hock, Gérard Waeber, Martin Preisig, Peter Vollenweider

**Affiliations:** 1 Institute of Social and Preventive Medicine (IUMSP), CHUV and Faculty of Biology and Medicine, Lausanne, Switzerland; 2 Department of Medicine, Internal Medicine, CHUV and Faculty of Biology and Medicine, Lausanne, Switzerland; 3 Department of Medicine, University of Zürich, Zürich, Switzerland; 4 Department of Psychiatry, University of Geneva, Geneva, Switzerland; 5 Department of Community Medicine, University of Geneva, Geneva, Switzerland; 6 Cytolab, Dällikon, Switzerland; 7 Division of Psychosomatic Medicine, Bern University Hospital Inselspital, and University of Bern, Bern, Switzerland; 8 Department of Psychiatry, University Hospital Zürich, Zürich, Switzerland; 9 Department of Psychiatry, CHUV, Lausanne, Switzerland; University of Tor Vergata, Italy

## Abstract

**Objective:**

to assess the levels and determinants of interleukin (IL)-1β, IL-6, tumour necrosis factor (TNF)-α and C-reactive protein (CRP) in a healthy Caucasian population.

**Methods:**

population sample of 2884 men and 3201 women aged 35 to 75. IL-1β, IL-6 and TNF-α were assessed by a multiplexed particle-based flow cytometric assay and CRP by an immunometric assay.

**Results:**

Spearman rank correlations between duplicate cytokine measurements (N = 80) ranged between 0.89 and 0.96; intra-class correlation coefficients ranged between 0.94 and 0.97, indicating good reproducibility. Among the 6085 participants, 2289 (37.6%), 451 (7.4%) and 43 (0.7%) had IL-1β, IL-6 and TNF-α levels below detection limits, respectively. Median (interquartile range) for participants with detectable values were 1.17 (0.48–3.90) pg/ml for IL-1β; 1.47 (0.71–3.53) pg/ml for IL-6; 2.89 (1.82–4.53) pg/ml for TNF-α and 1.3 (0.6–2.7) ng/ml for CRP. On multivariate analysis, greater age was the only factor inversely associated with IL-1β levels. Male sex, increased BMI and smoking were associated with greater IL-6 levels, while no relationship was found for age and leisure-time PA. Male sex, greater age, increased BMI and current smoking were associated with greater TNF-α levels, while no relationship was found with leisure-time PA. CRP levels were positively related to age, BMI and smoking, and inversely to male sex and physical activity.

**Conclusion:**

Population-based levels of several cytokines were established. Increased age and BMI, and to a lesser degree sex and smoking, significantly and differentially impact cytokine levels, while leisure-time physical activity has little effect.

## Introduction

Proinflammatory cytokines, such as interleukin (IL)-1β, IL-6 and tumor necrosis factor (TNF)-α and the acute phase reactant C-reactive protein (CRP) have important effects in inflammation and atherosclerosis. Elevated levels of these inflammatory biomarkers have been associated with an increased risk of developing incident coronary heart disease [1]–[3].

Several studies have shown that cytokine levels can be mediated by several lifestyle factors such as smoking [4] and physical activity [4]–[6]. Still, contrary to the considerable data regarding the contribution of cytokines to atherothrombotic diseases, little information is available regarding the distribution of cytokine levels and their determinants within a population-based sample [7], [8].

Hence, we used the data from the large, population-based CoLaus study to 1) assess cytokine levels in an apparently healthy, population-based Caucasian adult sample and 2) assess the independent effects of sex, age, BMI, smoking and physical activity on cytokine levels. To our knowledge, this is currently the largest population study that has cytokines levels measured.

## Methods

### Ethics statement

The CoLaus Study was approved by the Institutional Ethics Committee of the University of Lausanne (decision 19 February 2003, protocol number 16/03). Written informed consent was obtained from all participants.

### Recruitment

The CoLaus Study is a cross-sectional study aimed at assessing the prevalence of CVD risk factors as the molecular determinants of CVD in the Caucasian population of Lausanne, Switzerland, a town of 117,161 inhabitants, of which 79,420 are of Swiss nationality. The sampling procedure of the CoLaus Study has previously been described [9]. Recruitment began in June 2003 and ended in May 2006. Participation rate was 41%.

All participants attended the outpatient clinic of the University Hospital of Lausanne in the morning after an overnight fast. Data were collected by trained field interviewers in a single visit lasting about 60 min. No information regarding revenues or social deprivation was collected.

### Lifestyle and clinical data

Participants were classified as never, current, or former smokers. A participant was considered as physically active if he/she reported practicing at least 2 hours of leisure-time physical activity per week.

Body weight and height were measured with participants standing without shoes in light indoor clothes. Body weight was measured in kilograms to the nearest 100 g using a Seca® scale, regularly calibrated. Height was measured to the nearest 5 mm using a Seca® height gauge. Overweight was defined as a BMI ≥25 and <30 kg.m^−2^; obesity was defined as a BMI ≥30 kg.m^−2^.

### Cytokine measurement

Venous blood samples (50 mL) were drawn in the fasting state and allowed to clot. Serum was preferred to plasma as it has been shown that different anticoagulants may affect absolute cytokine levels differently [10], [11]. High sensitive CRP (hs-CRP) was assessed by immunoassay and latex HS (IMMULITE 1000–High, Diagnostic Products Corporation, LA, CA, USA) with maximum intra- and interbatch coefficients of variation of 1.3% and 4.6%, respectively. Serum samples were kept at −80°C before assessment of IL-1β, IL-6, and TNF-α and sent in dry ice to the laboratory. Levels of these cytokines were measured using a multiplexed particle-based flow cytometric cytokine assay [12]. This methodology yields cytokine concentrations which correlate well with those obtained by other methods such as ELISA [13] (for a review, see [14]). Milliplex kits were purchased from Millipore (Zug, Switzerland). The procedures closely followed the manufacturer's instructions. The analysis was conducted using a conventional flow cytometer (FC500 MPL, BeckmanCoulter, Nyon, Switzerland). Lower limits of detection (LOD) for IL-1β, IL-6 and TNF-α were 0.2 pg/ml. A good agreement between signal and cytokine was found within the assay range (R^2^≥0.99). Intra and inter-assay coefficients of variation were respectively 15% and 16.7% for IL-1β, 16.9% and 16.1% for IL-6 and 12.5% and 13.5% for TNF-α. For quality control, repeated measurements were conducted in 80 subjects randomly drawn from the initial sample.

### Statistical analysis

Statistical analysis was conducted using SAS v.9.2 (SAS Inc, Cary, NC, USA). Reproducibility between the first and the second measurement was assessed by Spearman nonparametric correlation, intraclass correlation coefficients, Lin's concordance correlation and Bland-Altman plots. Lin's concordance correlation measures how well a new set of observations reproduces an original set and has been reported to be more appropriate than other indices for measuring agreement when the variable of interest is continuous. Quantitative variables (apart from inflammatory biomarkers) were expressed as mean ± standard deviation and qualitative variables as number of participants and (percentage). Biomarkers were presented as median and (interquartile range) of measured values, percentage of values below LOD and percentage of values within each quartile. Undetectable values were included in the first quartile. Between groups comparisons were performed using Student t-test or Kruskall-Wallis nonparametric test for quantitative and chi-square test for qualitative variables. The relationships between biomarker values (excluding undetectable ones) and selected quantitative variables (i.e. age and BMI) were assessed using Spearman's nonparametric correlation; similar analyses were performed replacing values below LOD by a) half the limit of detection [15] and b) multiple imputation of missing data using a Markov Chain Monte Carlo method [16] and five imputation sets. Multivariate analysis was conducted by multivariate linear regression using log-transformed cytokine values as dependent variable, a method used elsewhere [4]. Two models were applied: the first using only measured data, the second replacing values below LOD by half the limit of detection. The results were expressed as slope and (standard error). We also used multivariate logistic regression to assess the likelihood of being in the topmost quartile compared to the other three quartiles as well as being in the topmost vs. the lowest quartile of cytokine distribution. Results of the logistic analysis were presented as Odds-ratio (OR) and (95% confidence interval). Statistical significance was considered for p<0.05.

## Results

### Clinical characteristics of participants

Of the 6,188 initial participants, 6,085 (98.3%, 2884 men and 3201 women) could be assessed for inflammatory biomarkers while for the remaining 103 participants (1.7%) no blood samples were available. Compared to women, men were older (52.6±10.8 vs. 53.5±10.7 years, p<0.001), had a higher BMI (26.6±4.0 vs. 25.1±4.8 kg/m^2^, p<0.001) and smoked more (32.3%, 38.6% and 29.1% for never, former and current smokers, respectively, vs. 47.3, 28.0 and 24.7%, p<0.001). Conversely, leisure-time physical activity was similar between sexes (men: 64.2%, women: 65.0%; p = 0.51).

### Reproducibility of cytokine measurements

Spearman rank correlations (N = 80) between duplicate measurements were 0.914, 0.961 and 0.891 for IL-1β, IL-6 and TNF-α (all p<0.001), respectively, while Lin's correlation coefficients were 0.969, 0.971 and 0.945 and intra-class correlation coefficients were 0.970, 0.972 and 0.946 for IL-1β, IL-6 and TNF-α, respectively (all p<0.001), indicating a good reproducibility. Bland-Altman plots also showed good average agreement (not shown).

### Distribution of cytokine levels

Among the 6085 participants, 2289 (37.6%), 451 (7.4%) and 43 (0.7%) had IL-1ß, Il-6 and TNF-α levels below LOD, respectively. The distribution of measured IL-1β, IL-6, TNF-α and hs-CRP levels according to different criteria is summarized in **Tables 1** **,** **2** **,** **3** **and** **4**, respectively.

**Table 1:** Interleukin-1β distribution according to different parameters.

	All	Dosable	Median,pg/ml	% below		% in quartile		
	N	N	(IQR)	LOD	1	2	3	4
All subjects	6085	3796	1.17(0.48–3.90)	37.6	37.6	20.7	20.5	21.2
Sex								
Men	2884	1746	1.08(0.45–3.96)	39.5	39.5	21.7	18.9	20.0
Women	3201	2050	1.28(0.52–3.79)	36.0	36.0	19.8	22.0	22.2
Test			4.33*	7.93**		18.37***		
Age group (years)								
[35–44]	1714	1172	1.32(0.54–4.30)	31.6	31.6	20.8	22.6	25.0
[45–54]	1734	1103	1.26(0.48–3.98)	36.4	36.4	20.7	20.3	22.6
[55–64]	1677	1007	1.02(0.44–3.27)	40.0	40.0	21.7	20.2	18.2
[65–75]	960	514	1.13(0.45–3.55)	46.5	46.5	18.9	17.7	17.0
Test			14.61**	63.24***		77.72***		
BMI categories								
Normal	2925	1874	1.32(0.53–4.09)	35.9	35.9	19.3	22.0	22.8
Overweight	2222	1357	1.06(0.45–3.90)	38.9	38.9	22.2	18.5	20.3
Obese	938	565	1.03(0.45–2.91)	39.8	39.8	21.4	20.8	18.0
Test			10.35*	7.02*		26.21***		
Smoking status								
Never	2445	1536	1.17(0.51–3.70)	37.2	37.2	20.2	21.7	20.9
Former	2009	1229	1.10(0.45–3.82)	38.8	38.8	21.7	19.2	20.3
Current	1631	1031	1.27(0.49–4.46)	36.8	36.8	20.2	20.4	22.6
Test			4.49^NS^	1.93^NS^		8.57^NS^		
Leisure-time PA								
No	2156	1369	1.19(0.47–3.82)	36.5	36.5	21.0	21.6	20.9
Yes	3929	2427	1.17(0.49–3.93)	38.2	38.2	20.5	20.0	21.3
Test			0.01^NS^	1.77^NS^		3.13^NS^		

Results are expressed as median and (interquartile range, IQR) for values over detection level, and as % (all subjects). BMI, body mass index; CVD, cardiovascular disease; LOD, limits of detection; PA, physical activity. Statistical analysis by Kruskall-Wallis nonparametric test (for medians) and by chi-square (for percentages):

NS , not significant;

*, p<0.05;

**, p<0.01;

***, p<0.001.

**Table 2:** Interleukin-6 distribution according to different parameters.

	All	Dosable	Median,pg/ml	% below		% in quartile		
	N	N	(IQR)	LOD	1	2	3	4
All subjects	6085	5634	1.47(0.71–3.53)	7.4	25.1	24.8	25.1	25.0
Sex								
Men	2884	2697	1.59(0.77–3.84)	6.5	22.3	24.4	26.0	27.3
Women	3201	2937	1.37(0.67–3.23)	8.2	27.5	25.1	24.4	23.0
Test			19.62***	6.87**		29.17***		
Age group (years)								
[35–44]	1714	1550	1.38(0.65–3.51)	9.6	29.5	23.6	22.6	24.3
[45–54]	1734	1594	1.41(0.69–3.54)	8.1	26.2	25.6	23.3	24.9
[55–64]	1677	1579	1.52(0.71–3.40)	5.8	23.8	25.0	26.3	24.9
[65–75]	960	911	1.70(0.90–3.68)	5.1	17.3	24.9	30.8	27.0
Test			18.36***	26.18***		61.68***		
BMI categories								
Normal	2925	2652	1.36(0.64–3.46)	9.3	29.6	24.0	22.3	24.1
Overweight	2222	2075	1.45(0.73–3.37)	6.6	23.8	26.2	25.7	24.4
Obese	938	907	1.96(0.99–4.04)	3.3	14.1	23.6	32.6	29.7
Test			47.58***	40.85***		113.74***		
Smoking status								
Never	2445	2235	1.32(0.65–3.11)	8.6	28.6	25.6	23.7	22.1
Former	2009	1853	1.50(0.71–3.61)	7.8	25.6	24.4	24.6	25.4
Current	1631	1546	1.73(0.86–4.06)	5.2	19.2	24.0	27.8	29.0
Test			39.69***	16.81***		61.20***		
Leisure-time PA								
No	2156	2027	1.63(0.77–3.68)	6.0	22.0	24.2	27.3	26.5
Yes	3929	3607	1.41(0.69–3.42)	8.2	26.7	25.1	23.9	24.3
Test			11.03***	9.93**		21.86***		

Results are expressed as median and (interquartile range, IQR) for values over detection level, and as % (all subjects). BMI, body mass index; CVD, cardiovascular disease; LOD, limits of detection; PA, physical activity. Statistical analysis by Kruskall-Wallis nonparametric test (for medians) and by chi-square (for percentages):

**, p<0.01;

***, p<0.001.

**Table 3:** Tumor Necrosis Factor-α distribution according to different parameters.

	All	Dosable	Median,pg/ml	% below		% in quartile		
	N	N	(IQR)	LOD	1	2	3	4
All subjects	6085	6042	2.89(1.82–4.53)	0.7	25.2	24.7	25.2	24.9
Sex								
Men	2884	2865	3.05(1.90–4.65)	0.7	23.1	23.9	27.1	26.0
Women	3201	3177	2.75(1.73–4.42)	0.7	27.2	25.5	23.4	23.9
Test			22.59***	0.18^NS^		22.89***		
Age group (years)								
[35–44]	1714	1701	2.59(1.66–4.08)	0.8	29.7	27.3	22.0	21.1
[45–54]	1734	1719	2.87(1.76–4.44)	0.9	26.9	23.8	25.3	24.0
[55–64]	1677	1667	3.04(1.94–4.63)	0.6	22.5	24.3	27.4	25.8
[65–75]	960	955	3.31(2.11–5.18)	0.5	19.1	22.6	26.8	31.6
Test			74.70***	1.45^NS^		81.07***		
BMI categories								
Normal	2925	2901	2.70(1.71–4.21)	0.8	27.9	26.2	24.2	21.7
Overweight	2222	2209	2.97(1.85–4.61)	0.6	24.5	23.8	25.8	25.9
Obese	938	932	3.41(2.13–5.27)	0.6	18.7	22.5	26.8	32.1
Test			66.54***	1.07^NS^		64.78***		
Smoking status								
Never	2445	2430	2.79(1.76–4.42)	0.6	26.5	25.9	24.1	23.4
Former	2009	1988	2.97(1.84–4.54)	1.0	24.9	23.8	26.4	24.9
Current	1631	1624	3.00(1.87–4.80)	0.4	23.7	24.0	25.3	27.0
Test			11.39**	5.38^NS^		13.03*		
Leisure-time PA								
No	2156	2140	2.99(1.85–4.76)	0.7	25.0	23.1	25.1	26.9
Yes	3929	3902	2.85(1.81–4.45)	0.7	25.4	25.6	25.3	23.8
Test			5.68*	0.06^NS^		9.24*		

Results are expressed as median and (interquartile range, IQR) for values over detection level, and as % (all subjects). BMI, body mass index; CVD, cardiovascular disease; LOD, limits of detection; PA, physical activity. Statistical analysis by Kruskall-Wallis nonparametric test (for medians) and by chi-square (for percentages):

NS , not significant;

*, p<0.05;

**, p<0.01;

***, p<0.001.

**Table 4:** High sensitive-CRP distribution according to different parameters.

	All	Dosable	Median,pg/ml		% in quartile		
	N	N	(IQR)	1	2	3	4
All subjects	6085	6084	1.3 (0.6–2.7)	21.9	29.8	22.4	25.8
Sex							
Men	2884	2883	1.2 (0.6–2.6)	22.0	31.1	23.3	23.6
Women	3201	3201	1.3 (0.6–2.9)	21.9	28.7	21.6	27.8
Test			6.16**		15.15**		
Age group (years)							
[35–44]	1714	1713	1.0 (0.4–2.2)	31.3	29.9	17.3	21.5
[45–54]	1734	1734	1.1 (0.6–2.4)	24.0	32.8	20.9	22.3
[55–64]	1677	1677	1.5 (0.7–3.1)	15.5	29.6	25.3	29.5
[65–75]	960	960	1.8 (0.9–3.4)	12.6	24.8	29.2	33.4
Test			226.8***		254.3***		
BMI categories							
Normal	2925	2924	0.8 (0.4–1.7)	33.9	33.8	17.7	14.6
Overweight	2222	2222	1.6 (0.8–3.1)	13.4	30.4	26.6	29.6
Obese	938	938	2.8 (1.5–5.5)	4.9	16.2	27.1	51.8
Test			929.2***		916.2***		
Smoking status							
Never	2445	2445	1.2 (0.6–2.6)	23.0	30.5	21.6	24.8
Former	2009	2009	1.3 (0.6–2.7)	21.9	30.1	22.8	25.3
Current	1631	1630	1.4 (0.7–2.9)	20.4	28.5	23.2	28.0
Test			13.6**		9.81^NS^		
Leisure-time PA							
No	2156	2156	1.6 (0.7–3.5)	16.6	27.0	24.4	32.0
Yes	3929	3928	1.1 (0.6–2.4)	24.8	31.4	21.4	22.4
Test			119.88***		106.94***		

Results are expressed as median and (interquartile range, IQR) for values over detection level, and as % (all subjects). BMI, body mass index; CVD, cardiovascular disease; LOD, limits of detection; PA, physical activity. Statistical analysis by Kruskall-Wallis nonparametric test (for medians) and by chi-square (for percentages):

NS , not significant;

**, p<0.01;

***, p<0.001.

For IL-1β, lower levels (and higher percentage of subjects below detection values) were found in men and with increasing age or BMI, while no differences were found between the three smoking groups or with increasing leisure-time physical activity (**Table 1**). IL-1β values were inversely related with age and BMI, and positively with IL-6 and TNF-α, while no relationship was found with CRP (**Table S6**). For IL-6, higher levels (and lower percentage of subjects below the LOD) were found in men, with increasing age and BMI, among current smokers and sedentary subjects (**Table 2**). Significant positive correlations were found between IL-6 and age, BMI, TNF-α and CRP (**Table S1**). For TNF-α, higher levels were found in men, with increasing age and BMI, among smokers and sedentary subjects (**Table 3**). As for IL-6, significant correlations were found between TNF-α values and age, BMI and CRP (**Table S1**). For hs-CRP, higher levels were found in women, with increasing age and BMI and among smokers, while lower levels were found in subjects who reported leisure-time physical activity (**Table 4**). Positive relationships were found between hs-CRP values and age and BMI (**Table S1**).

The relationship of cytokine levels (including the proportion of undetectable values) with age differed considerably across biomarkers and by sex (**Figure S1**). IL-1β linearly decreased with age in both sexes, women having higher levels than men at all age groups. IL-6 levels tended to be higher at greater ages in both sexes, but the association with age was not linear and tended to be steeper in men than in women. TNF-α levels increased with age in men in a nearly linear manner, whereas the increase in women occurred mainly around the age of menopause. For hs-CRP, the age-related increase was linear in men and S-shaped in women.

### Multivariate analysis of the factors related with cytokine levels

The results of the multivariate linear regression analysis are summarized in **Table 5**. Multivariate analysis assessing the likelihood of being in the topmost a higher quartile compared to the lower other quartiles of cytokine distribution was performed using ordinal logistic regression. For IL-1β, older age were significantly, independently and inversely related with IL-1β levels, while no associations were found for the other variables. For IL-6, male sex, increased BMI and smoking status were independently and positively related with IL-6 levels, while no significant association was found for age and leisure-time physical activity. For TNF-α, male sex, older age, increased BMI and current smoking were positively related with TNF-α, while no association were found for leisure-time physical activity. Finally, for hs-CRP, increasing age, and BMI and current smoking were positively related while male sex and leisure-time physical activity were negatively associated with hs-CRP levels (**Table 5**). These findings were further confirmed by multivariate logistic regression modeling the likelihood of being in the highest vs. the others or the lowest quartiles, and including values below LOD in the lowest quartile (**Table S2 and Table S3**). Including values below LOD further showed an inverse association between male sex and IL-1β levels and a positive association between age and (**Table S2 and Table S3**).

**Table 5:** Multivariate linear regression using log-transformed values of cytokines.

	IL-1β	IL-6	TNF-α	CRP
Men(yes vs. no)	−0.0638(0.0495)	0.0756(0.0366)*	0.0517(0.0228)*	−0.2210(0.0261)***
Age	−0.0077(0.0023) ***	0.0029(0.0017)	0.0071(0.0011)***	0.0128(0.0012)***
BMI	−0.0074(0.0055)	0.0224(0.0041)***	0.0163(0.0026)***	0.0969(0.0029)***
Former smoker(yes vs. no)	0.0080(0.0572)	0.0965(0.0425)*	0.0297(0.0264)	−0.0030(0.0301)
Current smoker(yes vs. no)	0.1118(0.0605)	0.2524(0.0450)***	0.1165(0.0280)***	0.2327(0.0321)***
Leisure-time PA(yes vs. no)	−0.0035(0.0512)	−0.0276(0.0378)	−0.0063(0.0236)	−0.1520(0.0270)***

Results are expressed as slope and (standard error). BMI, body mass index; hs-CRP, high sensitive C reactive protein; IL-1β, interleukin-1β; IL-6, interleukin-6; PA, physical activity; TNF-α, tumor necrosis factor-α. Statistical analysis by linear regression on log-transformed cytokine values:

*, p<0.05;

***, p<0.001.

### Results after replacement of undetectable values

As a significant number of participants had cytokine levels below LOD, further analyses were conducted replacing values below LOD by half the LOD or using multivariate imputation as described. The results are summarized in supplemental tables 4 to 6. Overall, and as observed using measured values only, IL-1β values were inversely related with age and BMI, and positively with IL-6 and TNF-α, while no relationship was found with CRP (**Table S4 and Table S5**). Similarly, the results of the multivariate linear regression analysis were comparable with these using only measured data, with the exception of an inverse association between male sex and IL-1β levels and a positive association between age and IL-6 levels (**Table S6**), a finding also observed using multivariate logistic regression (**Table S2 and Table S3**).

## Discussion

There are few population studies providing information on cytokines [7], [17]. To our knowledge, this is one of the largest population-based studies which assessed the distributions and determinants of circulating inflammatory biomarkers. Our results thus provide important information regarding the distribution of levels of these biomarkers in the Caucasian adult population, which could serve as reference values for further studies.

The reproducibility of the IL-1β, IL-6 and TNF-α assays was adequate, with between-measurement correlation coefficients higher than 0.9 and a good reproducibility. The intra- and inter-batch CVs were also below the reference 20% threshold [18], although higher thresholds have been used for cytokine assessments [19]. All samples were kept at −80°C before assessment. It has been shown that IL-6 and TNF-α levels kept at −70°C correspond to the initial values [20]. Interestingly, the correlation coefficients between IL-1β, IL-6 and TNF-α found in this study were in close agreement with the values reported previously, even after missing value replacement [21], [22] and the IL-6 and TNF-α values obtained using this methodology were comparable to the literature (**Figure S2 and Figure S3**). Finally, the fact that the CoLaus study used the same methodology on the same platform for all samples at baseline is of importance, as it has been shown that the results of cytokine assessment can differ considerably between platforms [19]. Overall, our data indicate that the cytokine measurements used in this study are reproducible and provide values and relationships in agreement with the literature.

In this study, circa 38% of participants had IL-1β below detection levels, a value lower than reported previously [21], [23]. This difference cannot be solely attributed to a lower detection threshold (0.2 pg/ml) of the method used in this study, as it is actually higher than reported in other studies (0.1 pg/ml) [23]. Likely explanations include the use of plasma instead of serum [21]; different blood collection periods [24] or the type of anticoagulation used [21]. Overall, it would be helpful that each study reports the percentage of participants below detection levels as well as the method used (kit and serum or plasma samples), in order to adequately compare levels across studies.

To our knowledge, there has been little information regarding the factors influencing IL-1β levels at the population level. In this study, men had lower IL-1β levels than women, and this difference remained after multivariate adjustment. Hence, our results are not in agreement with previous studies suggesting that men have higher percentage of IL-1β secreting monocytes than women [25]. Increased age was also associated with lower IL-1β levels, and this difference persisted after multivariate adjustment. This is, to our knowledge, the first report providing the distribution of IL-1β by age and sex groups in the general population. Despite being a pro-inflammatory cytokine positively correlated with IL-6 and TNF-α, both of which increase with age, IL-1β levels were lower at older ages. Again, these findings do not confirm a previous study in which IL-1β levels were suggested to be similar between young and elderly subjects [22]. Contrary to a previous study [26], no relationship was found between personal history of CVD and IL-1β levels, possibly due to CVD treatment or to the fact that some participants presented their CVD event a long time ago. Conversely, the absence of relationship between IL-1β levels and leisure-time physical activity is in agreement with the literature [27]. Overall, our data indicate that IL-1β levels are positively, independently and significantly influenced by age and to a lesser degree by sex, but not by BMI, smoking or physical activity.

Men had higher IL-6 values than women, and this difference remained after multivariate adjustment, contradicting previous statements suggesting that the sex difference in IL-6 levels could be due to differences in adiposity [28]. Increased BMI and current smoking were also positively related with IL-6 levels, confirming previous findings [4]. Indeed, in healthy subjects, about 30% of circulating IL-6 originates from adipose tissue [29]. In agreement with some studies [30], but not with others [31], no significant independent relationship was found between age and IL-6 after multivariate adjustment. On bivariate analysis, lower IL-6 levels were found among participants who reported leisure-time physical activity, but this relationship became nonsignificant after multivariate analysis. Our results are in agreement with some studies [27], but not with others [6], suggesting that exercise reduces IL-6 independently from adiposity. Further, some authors have suggested that muscle contraction increases IL-6 levels, which then would act as an anti-inflammatory agent [32]. Overall, our data indicate that IL-6 levels are positively, independently and significantly influenced by sex, smoking status and increased BMI levels, while the effects of age and leisure-time (and overall) physical activity need further clarification.

Male sex and greater age, increased BMI and current smoking were independently and positively associated with TNF-α levels, a finding in agreement with the literature [33]. Contrary to some studies [5], but in agreement with others [27], no independent relationship between leisure-time physical activity and TNF-α levels was found. Again, it is possible that this relationship is mediated by exercise-induced changes in BMI, but further studies are needed to better assess this point. Overall, our data indicate that TNF-α levels are positively, independently and significantly influenced by male sex, age, smoking status and increased BMI levels, while the effects of and leisure-time physical activity need further clarification.

Significant positive relationships were found between hs-CRP levels, IL-6 and TNF-α, a finding already reported [34], although the strength of the relationship was lower in this study. Conversely, no relationships were found between hs-CRP and IL-1ß levels. CRP was positively and independently related with age, increased BMI and smoking, a finding reported previously [4]. Since adipose tissue can produce IL-6 [29], which in turn increases CRP production, it could be inferred that part of the relationship between BMI and hs-CRP could be mediated by high IL-6 levels; still, after adjusting for IL-6, the partial Spearman correlation between hs-CRP and BMI was virtually unchanged (0.398 instead of 0.408). The strength of the relationship between hs-CRP and BMI was also considerably higher than the relationships between BMI and the other cytokines, suggesting that the relationships between the different cytokines and BMI appear to be graded and rather complex. Finally, the negative relationship between hs-CRP and leisure-time physical activity is in agreement with some studies [6], [27] but not with others [4]. Overall, our data indicate that hs-CRP levels are positively related with age, increasing BMI and smoking, and negatively related with male sex and leisure-time physical activity.

This study has some limitations worth pointing out. The participation rate was low (41%), which might limit the generalization of the findings; however, this participation rate is similar to other epidemiological studies [35]. Also, no data on non-Caucasian participants were available; therefore our findings may not apply to other ethnicities. Further, only data from leisure-time physical activity was available. Hence, it is likely that this sole information may be not sufficient to detect any impact on levels of pro-inflammatory cytokines. Further studies with a better assessment of overall physical activity are needed to clarify the association between physical activity and pro-inflammatory cytokines. The major strength of our study is that we used a large, population-based sample representative of the Swiss population, and that a precise characterization of the participants was performed.

### Conclusion

In summary, we provide population-based reference levels of several cytokines; these levels could be used for comparison with other specific groups. Our results also indicate that, in this population-based sample, levels of inflammatory biomarkers of atherothrombotic risk seem robustly influenced by age and increased BMI and to a lesser degree by sex and smoking, while the lower effect of leisure-time physical activity awaits further clarification.

## Supporting Information

Figure S1 Serum levels of interleukin-1β (IL-1β, panel A), interleukin-6 (IL-6, panel B), tumor necrosis factor-α (TNF-α, panel C) and high sensitivity C-reactive protein (hs-CRP, panel D) by 5-year age groups, stratified by gender. Undetectable values were replaced by the midpoint between the lower detection value and zero. Results are expressed in pg/ml for IL-1β, Il-6 and TNF-α and in ng/ml for hs-CRP, and as median and interquartile range.
(TIF)

Figure S2 Comparison of interleukin-6 (IL-6) values between the current study and the literature. IL-6 results are expressed as median and interquartile range. The studies are referenced by the first author, the country and the number of subjects. Black color, plasma; blue color, serum. Data for the current study (red) was obtained using serum samples.
(TIF)

Figure S3 Comparison of tumor necrosis factor-α (TNF-α) values between the current study and the literature. TNF-α results are expressed as median and interquartile range. The studies are referenced by the first author, the country and the number of subjects. Black color, plasma; blue color, serum. Data for the current study (red) was obtained using serum samples.
(TIF)

Table S1 (DOC)

Table S2 (DOC)

Table S3 (DOC)

Table S4 (DOC)

Table S5 (DOC)

Table S6 (DOC)
