# Supplementary figures and images for: Levels and Determinants of Inflammatory Biomarkers in a Swiss Population-Based Sample (CoLaus Study)
Source: PLoS One. 2011 Jun 9;6(6):e21002. doi: 10.1371/journal.pone.0021002 (PMC3111463; doi:10.1371/journal.pone.0021002)

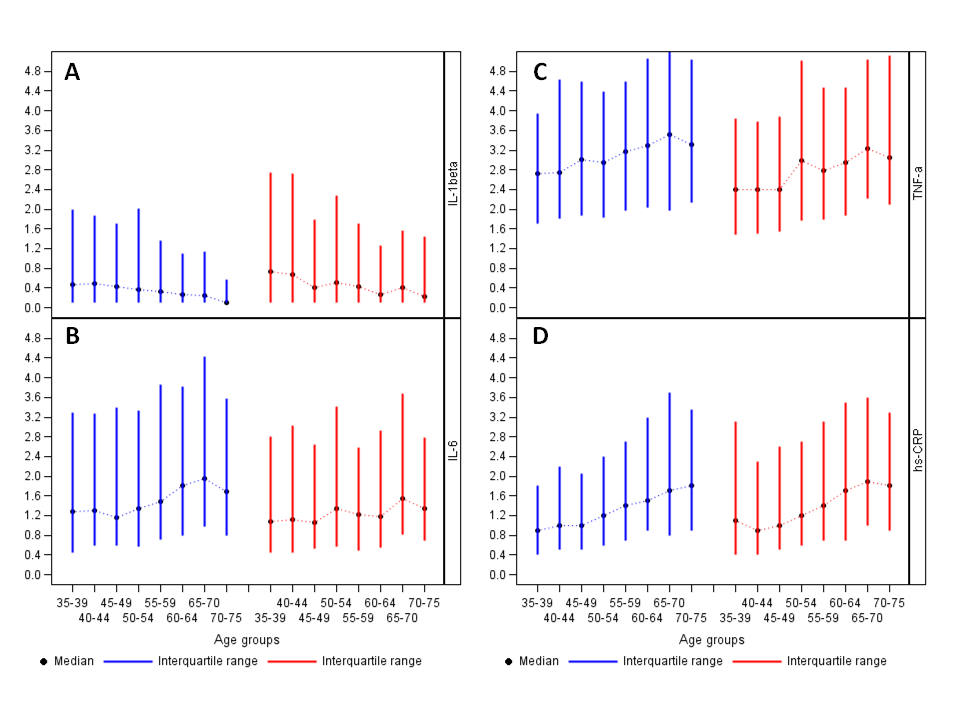

Supplement: Figure S1 — Serum levels of interleukin-1β (IL-1β, panel A), interleukin-6 (IL-6, panel B), tumor necrosis factor-α (TNF-α, panel C) and high sensitivity C-reactive protein (hs-CRP, panel D) by 5-year age groups, stratified by gender. Undetectable values were replaced by the midpoint between the lower detection value and zero. Results are expressed in pg/ml for IL-1β, Il-6 and TNF-α and in ng/ml for hs-CRP, and as median and interquartile range. (TIF) [file pone.0021002.s001.tif]

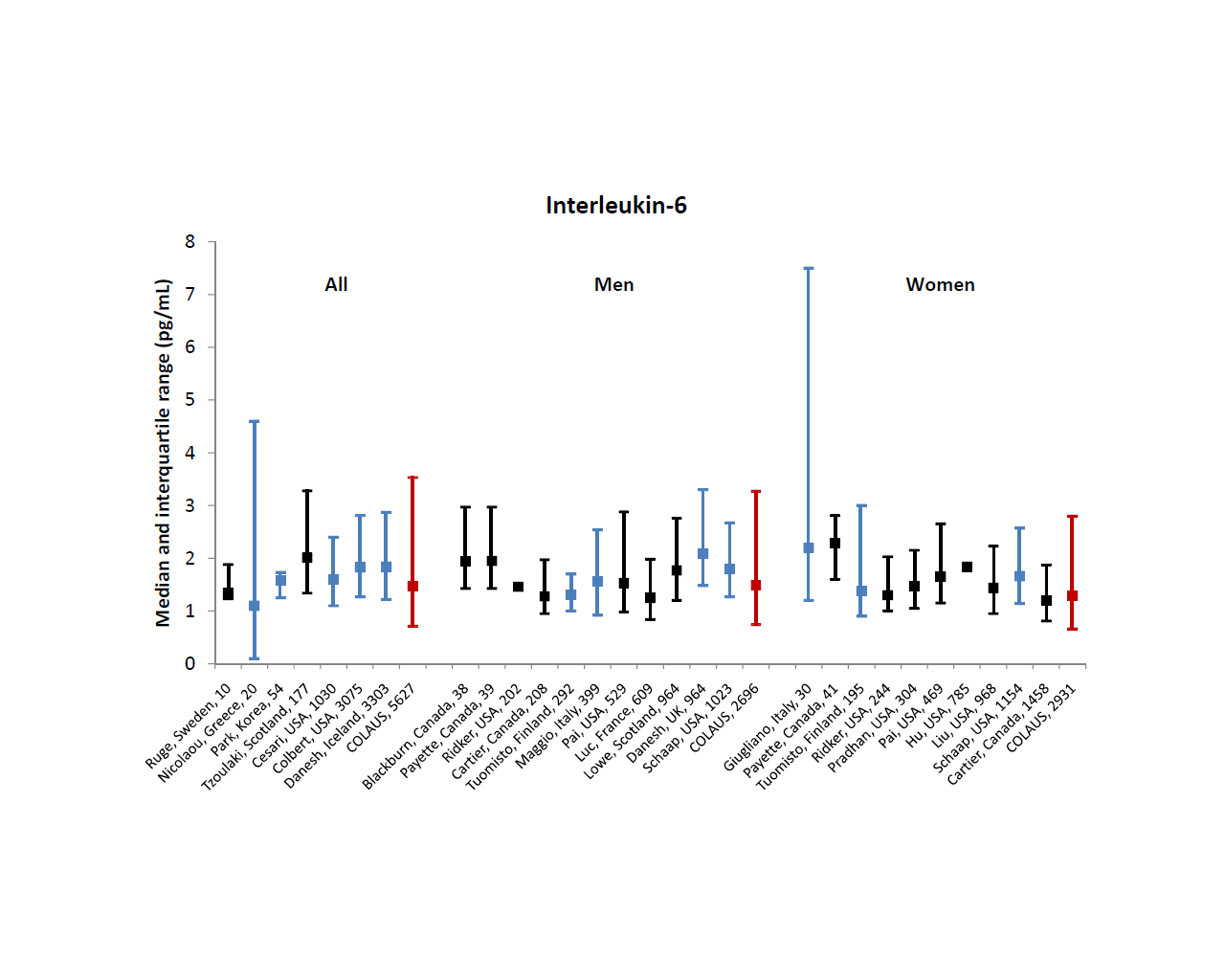

Supplement: Figure S2 — Comparison of interleukin-6 (IL-6) values between the current study and the literature. IL-6 results are expressed as median and interquartile range. The studies are referenced by the first author, the country and the number of subjects. Black color, plasma; blue color, serum. Data for the current study (red) was obtained using serum samples. (TIF) [file pone.0021002.s002.tif]

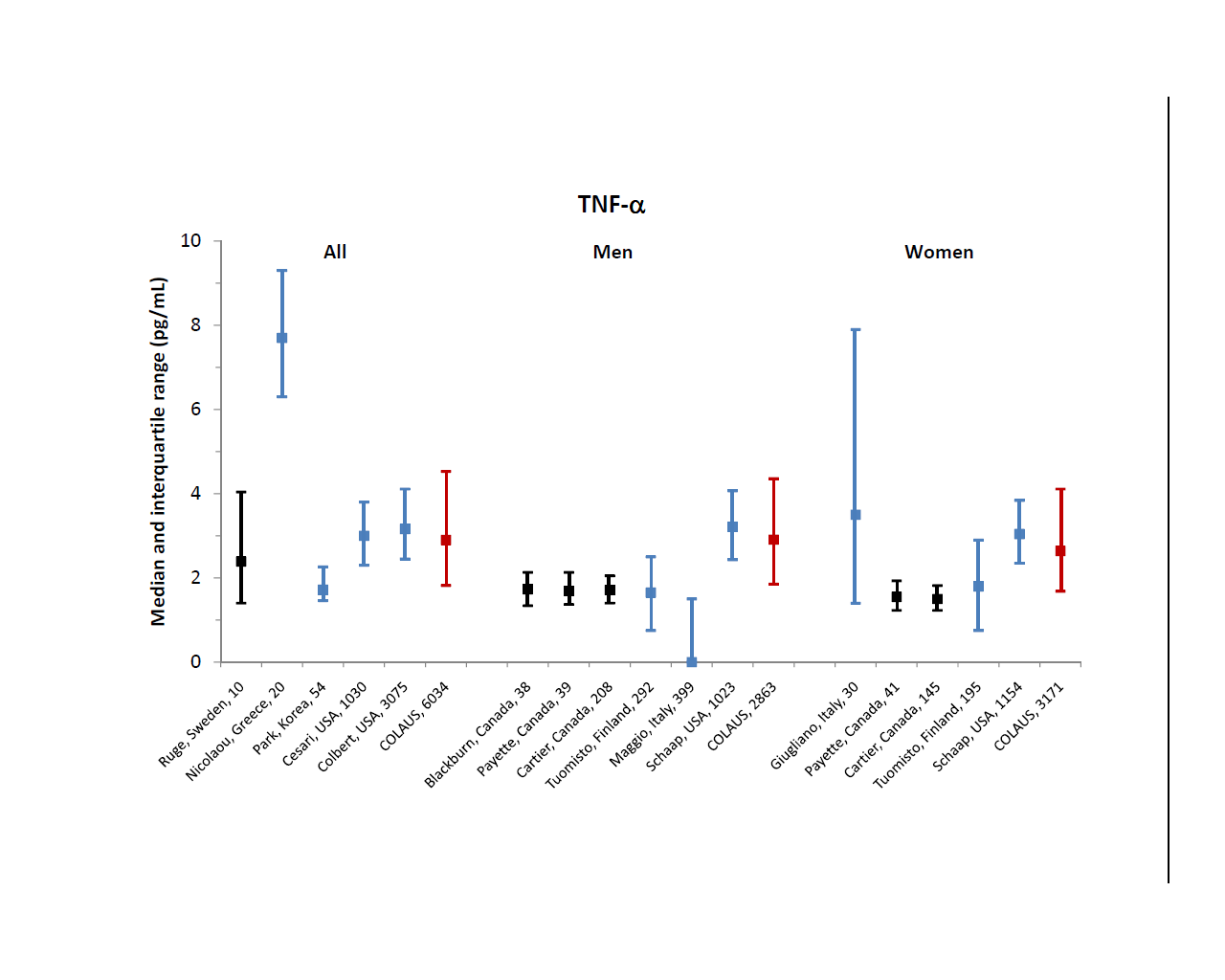

Supplement: Figure S3 — Comparison of tumor necrosis factor-α (TNF-α) values between the current study and the literature. TNF-α results are expressed as median and interquartile range. The studies are referenced by the first author, the country and the number of subjects. Black color, plasma; blue color, serum. Data for the current study (red) was obtained using serum samples. (TIF) [file pone.0021002.s003.tif]
