# Supplementary material for: Levels and Determinants of Inflammatory Biomarkers in a Swiss Population-Based Sample (CoLaus Study)
Source: PLoS One. 2011 Jun 9;6(6):e21002. doi: 10.1371/journal.pone.0021002 (PMC3111463; doi:10.1371/journal.pone.0021002)
Supplement: Table S1 — (DOC) [file pone.0021002.s004.doc]

**Table S1**: Spearman rank correlations between interleukins and selected variables

|  | **IL-1β** | **IL-6** | **TNF-α** | **hs-CRP** |
| --- | --- | --- | --- | --- |
| Age | -0.063 | 0.053 | 0.118 | 0.194 |
| p-value | <0.001 | <0.001 | <0.001 | <0.001 |
| Sample size | 3796 | 5634 | 6042 | 6084 |
| BMI | -0.056 | 0.089 | 0.122 | 0.408 |
| p-value | <0.001 | <0.001 | <0.001 | <0.001 |
| Sample size | 3795 | 5633 | 6041 | 6083 |
| IL-1β |  | 0.358 | 0.305 | -0.020 |
| p-value |  | <0.001 | <0.001 | 0.21 |
| Sample size |  | 3612 | 3774 | 3796 |
| IL-6 |  |  | 0.345 | 0.181 |
| p-value |  |  | <0.001 | <0.001 |
| Sample size |  |  | 5596 | 5633 |
| TNF-α |  |  |  | 0.119 |
| p-value |  |  |  | <0.001 |
| Sample size |  |  |  | 6041 |

BMI, body mass index; hs-CRP, high sensitive C reactive protein; IL-1β, interleukin-1β; IL-6, interleukin-6; TNF-α, tumor necrosis factor-α.
