# Supplementary material for: Levels and Determinants of Inflammatory Biomarkers in a Swiss Population-Based Sample (CoLaus Study)
Source: PLoS One. 2011 Jun 9;6(6):e21002. doi: 10.1371/journal.pone.0021002 (PMC3111463; doi:10.1371/journal.pone.0021002)
Supplement: Table S2 — (DOC) [file pone.0021002.s005.doc]

**Table S2**: logistic regression modeling the likelihood of being in the highest quartile of cytokine level vs. the three others.

|  | **IL-1β** | **IL-6** | **TNF-α** | **CRP** |
| --- | --- | --- | --- | --- |
| Gender |  |  |  |  |
| Women | 1 (ref.) | 1 (ref.) | 1 (ref.) | 1 (ref.) |
| Men | 0.87 (0.77-0.99) | 1.22 (1.08-1.38) | 1.07 (0.95-1.21) | 0.62 (0.55-0.70) |
| Age group |  |  |  |  |
| [35-44] | 1 (ref.) | 1 (ref.) | 1 (ref.) | 1 (ref.) |
| [45-54] | 0.88 (0.76-1.04) | 1.02 (0.87-1.19) | 1.15 (0.98-1.35) | 0.90 (0.76-1.07) |
| [55-64] | 0.68 (0.58-0.81) | 1.03 (0.88-1.21) | 1.25 (1.06-1.47) | 1.20 (1.01-1.41) |
| [65-75] | 0.63 (0.52-0.78) | 1.17 (0.97-1.41) | 1.68 (1.40-2.01) | 1.48 (1.23-1.79) |
| P-value for trend | 0.002 | 0.12 | <0.001 | <0.001 |
| BMI status |  |  |  |  |
| Normal | 1 (ref.) | 1 (ref.) | 1 (ref.) | 1 (ref.) |
| Overweight | 0.94 (0.81-1.08) | 0.96 (0.84-1.10) | 1.19 (1.04-1.36) | 2.64 (2.28-3.04) |
| Obese | 0.82 (0.68-1.00) | 1.30 (1.09-1.54) | 1.59 (1.35-1.89) | 6.52 (5.48-7.75) |
| P-value for trend | 0.19 | <0.001 | <0.001 | <0.001 |
| Smoking status |  |  |  |  |
| Never | 1 (ref.) | 1 (ref.) | 1 (ref.) | 1 (ref.) |
| Former | 1.01 (0.87-1.17) | 1.15 (1.00-1.32) | 1.03 (0.90-1.19) | 1.05 (0.91-1.22) |
| Current | 1.08 (0.93-1.26) | 1.42 (1.23-1.65) | 1.26 (1.08-1.46) | 1.46 (1.25-1.70) |
| P-value for trend | 0.43 | 0.006 | 0.02 | <0.001 |
| Leisure-time PA |  |  |  |  |
| No | 1 (ref.) | 1 (ref.) | 1 (ref.) | 1 (ref.) |
| Yes | 1.00 (0.88-1.14) | 0.94 (0.83-1.06) | 0.91 (0.81-1.04) | 0.77 (0.68-0.88) |

Results are expressed as Odds ratio and (95% confidence interval). BMI, body mass index; hs-CRP, high sensitive C reactive protein; IL-1β, interleukin-1β; IL-6, interleukin-6; PA, physical activity; TNF-α, tumor necrosis factor-α. Statistical analysis by logistic regression.
