# Supplementary material for: Levels and Determinants of Inflammatory Biomarkers in a Swiss Population-Based Sample (CoLaus Study)
Source: PLoS One. 2011 Jun 9;6(6):e21002. doi: 10.1371/journal.pone.0021002 (PMC3111463; doi:10.1371/journal.pone.0021002)
Supplement: Table S3 — (DOC) [file pone.0021002.s006.doc]

**Table S3**: logistic regression modeling the likelihood of being in the highest quartile of cytokine level vs. the lowest.

|  | **IL-1β** | **IL-6** | **TNF-α** | **CRP** |
| --- | --- | --- | --- | --- |
| Gender |  |  |  |  |
| Women | 1 (ref.) | 1 (ref.) | 1 (ref.) | 1 (ref.) |
| Men | 0.81 [0.70 - 0.94] | 1.36 [1.17 - 1.58] | 1.21 [1.04 - 1.40] | 0.59 [0.49 - 0.70] |
| Age group |  |  |  |  |
| [35-44] | 1 (ref.) | 1 (ref.) | 1 (ref.) | 1 (ref.) |
| [45-54] | 0.79 [0.66 - 0.95] | 1.09 [0.90 - 1.32] | 1.22 [1.01 - 1.48] | 1.22 [0.98 - 1.53] |
| [55-64] | 0.58 [0.48 - 0.71] | 1.20 [0.98 - 1.46] | 1.54 [1.26 - 1.87] | 2.03 [1.61 - 2.56] |
| [65-75] | 0.47 [0.38 - 0.59] | 1.88 [1.47 - 2.39] | 2.26 [1.79 - 2.85] | 3.16 [2.38 - 4.19] |
| P-value for trend | <0.001 | <0.001 | <0.001 | <0.001 |
| BMI status |  |  |  |  |
| Normal | 1 (ref.) | 1 (ref.) | 1 (ref.) | 1 (ref.) |
| Overweight | 0.93 [0.79 - 1.08] | 1.13 [0.96 - 1.34] | 1.24 [1.05 - 1.46] | 5.22 [4.31 - 6.33] |
| Obese | 0.82 [0.66 - 1.02] | 2.43 [1.91 - 3.09] | 2.02 [1.62 - 2.53] | 23.7 [17.0 - 33.2] |
| P-value for trend | 0.27 | <0.001 | <0.001 | <0.001 |
| Smoking status |  |  |  |  |
| Never | 1 (ref.) | 1 (ref.) | 1 (ref.) | 1 (ref.) |
| Former | 1 [0.85 - 1.18] | 1.19 [1.01 - 1.42] | 1.03 [0.87 - 1.23] | 1.05 [0.85 - 1.29] |
| Current | 1.06 [0.89 - 1.26] | 2.01 [1.67 - 2.43] | 1.39 [1.16 - 1.68] | 1.90 [1.53 - 2.37] |
| P-value for trend | 0.57 | <0.001 | <0.005 | <0.001 |
| Leisure-time PA |  |  |  |  |
| No | 1 (ref.) | 1 (ref.) | 1 (ref.) | 1 (ref.) |
| Yes | 0.95 [0.82 - 1.10] | 0.87 [0.74 - 1.02] | 0.98 [0.84 - 1.14] | 0.67 [0.55 - 0.80] |

Results are expressed as Odds ratio and (95% confidence interval). BMI, body mass index; hs-CRP, high sensitive C reactive protein; IL-1β, interleukin-1β; IL-6, interleukin-6; PA, physical activity; TNF-α, tumor necrosis factor-α. Statistical analysis by logistic regression.
