# Supplementary material for: Levels and Determinants of Inflammatory Biomarkers in a Swiss Population-Based Sample (CoLaus Study)
Source: PLoS One. 2011 Jun 9;6(6):e21002. doi: 10.1371/journal.pone.0021002 (PMC3111463; doi:10.1371/journal.pone.0021002)
Supplement: Table S4 — (DOC) [file pone.0021002.s007.doc]

**Table S4**: Spearman rank correlations between interleukins and selected variables, all subjects (n=6085), replacing missing values by half the LOD.

|  | **IL-1β** | **IL-6** | **TNF-α** | **hs-CRP** |
| --- | --- | --- | --- | --- |
| Age | -0.112 | 0.076 | 0.118 | 0.194 |
| p-value | <0.0001 | <0.0001 | <0.0001 | <0.0001 |
| BMI | -0.059 | 0.115 | 0.122 | 0.408 |
| p-value | <0.0001 | <0.0001 | <0.0001 | <0.0001 |
| IL-1β |  | 0.394 | 0.309 | -0.021 |
| p-value |  | <0.0001 | <0.0001 | 0.098 |
| IL-6 |  |  | 0.392 | 0.205 |
| p-value |  |  | <0.0001 | <.0001 |
| TNF-α |  |  |  | 0.118 |
| p-value |  |  |  | <0.0001 |

BMI, body mass index; hs-CRP, high sensitive C reactive protein; IL-1β, interleukin-1β; IL-6, interleukin-6; TNF-α, tumor necrosis factor-α.
