# Supplementary material for: Levels and Determinants of Inflammatory Biomarkers in a Swiss Population-Based Sample (CoLaus Study)
Source: PLoS One. 2011 Jun 9;6(6):e21002. doi: 10.1371/journal.pone.0021002 (PMC3111463; doi:10.1371/journal.pone.0021002)
Supplement: Table S5 — (DOC) [file pone.0021002.s008.doc]

**Table S5: Spearman rank correlations between interleukins and selected variables, all subjects (n=6085), replacing missing values by multivariate imputation. The values are average of 5 imputations.**

|  | **IL-1β** | **IL-6** | **TNF-α** | **hs-CRP** |
| --- | --- | --- | --- | --- |
| Age | -0.023 | 0.045 | 0.117 | 0.194 |
| BMI | -0.018 | 0.075 | 0.121 | 0.408 |
| IL-1β |  | 0.156 | 0.158 | -0.001 |
| IL-6 |  |  | 0.288 | 0.152 |
| TNF-α |  |  |  | 0.117 |

BMI, body mass index; hs-CRP, high sensitive C reactive protein; IL-1β, interleukin-1β; IL-6, interleukin-6; TNF-α, tumor necrosis factor-α.
