# Supplementary material for: Levels and Determinants of Inflammatory Biomarkers in a Swiss Population-Based Sample (CoLaus Study)
Source: PLoS One. 2011 Jun 9;6(6):e21002. doi: 10.1371/journal.pone.0021002 (PMC3111463; doi:10.1371/journal.pone.0021002)
Supplement: Table S6 — (DOC) [file pone.0021002.s009.doc]

**Table S6**: linear regression using log-transformed values of cytokines, undetectable values replaced by 0.1.

|  | **IL-1β** | **IL-6** | **TNF-α** |
| --- | --- | --- | --- |
| Men  (yes vs. no) | -0.1411 (0.0462)** | 0.0992 (0.0392)* | 0.0547 (0.0239)* |
| Age | -0.0171 (0.0021)*** | 0.0069 (0.0018)*** | 0.0073 (0.0011)*** |
| BMI | -0.008 (0.0052) | 0.0337 (0.0044)*** | 0.0173 (0.0027)*** |
| Former smoker  (yes vs. no) | 0.0041 (0.0533) | 0.0949 (0.0452)* | 0.013 (0.0275) |
| Current smoker  (yes vs. no) | 0.0651 (0.0568) | 0.3424 (0.0482)*** | 0.123 (0.0293)*** |
| Leisure-time PA  (yes vs. no) | -0.0602 (0.0478) | -0.0581 (0.0405) | -0.0004 (0.0247) |

Results are expressed as slope and (standard error)**.** *, p<0.05; **, p<0.01; ***, p<0.001
